# 4-Octyl itaconate attenuates glycemic deterioration by regulating macrophage polarization in mouse models of type 1 diabetes

**DOI:** 10.1186/s10020-023-00626-5

**Published:** 2023-03-14

**Authors:** Sunyue He, Yuchen Zhao, Guoxing Wang, Qiaofang Ke, Nan Wu, Lusi Lu, Jiahua Wu, Shuiya Sun, Weihua Jin, Wenjing Zhang, Jiaqiang Zhou

**Affiliations:** 1 Department of Endocrinology and Metabolism, Sir Run Run Shaw Hospital, Zhejiang University School of Medicine, Hangzhou, China; 2 Key Laboratory of Precision Medicine in Diagnosis and Monitoring Research of Zhejiang Province, Hangzhou, China; 3 College of Biotechnology and Bioengineering, Zhejiang University of Technology, Hangzhou, China

**Keywords:** 4-Octyl itaconate, Islet injury, Macrophage activation, Type 1 diabetes

## Abstract

**Background:**

Pancreatic beta cell dysfunction and activated macrophage infiltration are early features in type 1 diabetes pathogenesis. A tricarboxylic acid cycle metabolite that can strongly activate NF-E2-related factor 2 (Nrf2) in macrophages, itaconate is important in a series of inflammatory-associated diseases via anti-inflammatory and antioxidant properties. However, its role in type 1 diabetes is unclear. We used 4-octyl itaconate (OI), the cell-permeable itaconate derivate, to explore its preventative and therapeutic effects in mouse models of type 1 diabetes and the potential mechanism of macrophage phenotype reprogramming.

**Methods:**

The mouse models of streptozotocin (STZ)-induced type 1 diabetes and spontaneous autoimmune diabetes were used to evaluate the preventative and therapeutic effects of OI, which were performed by measuring blood glucose, insulin level, pro- and anti-inflammatory cytokine secretion, histopathology examination, flow cytometry, and islet proteomics. The protective effect and mechanism of OI were examined via peritoneal macrophages isolated from STZ-induced diabetic mice and co-cultured MIN6 cells with OI-pre-treated inflammatory macrophages in vitro. Moreover, the inflammatory status of peripheral blood mononuclear cells (PBMCs) from type 1 diabetes patients was evaluated after OI treatment.

**Results:**

OI ameliorated glycemic deterioration, increased systemic insulin level, and improved glucose metabolism in STZ-induced diabetic mice and non-obese diabetic (NOD) mice. OI intervention significantly restored the islet insulitis and beta cell function. OI did not alter the macrophage count but significantly downregulated the proportion of M1 macrophages. Additionally, OI significantly inhibited MAPK activation in macrophages to attenuate the macrophage inflammatory response, eventually improving beta cell dysfunction in vitro. Furthermore, we detected higher IL-1β production upon lipopolysaccharide stimulation in the PBMCs from type 1 diabetes patients, which was attenuated by OI treatment.

**Conclusions:**

These results provided the first evidence to date that OI can prevent the progression of glycemic deterioration, excessive inflammation, and beta cell dysfunction predominantly mediated by restricting macrophage M1 polarization in mouse models of type 1 diabetes.

**Supplementary Information:**

The online version contains supplementary material available at 10.1186/s10020-023-00626-5.

## Introduction

Type 1 diabetes is a progressive autoimmune disease characterized by inflammatory cell infiltration into islets and subsequently results in absolute insulin deficiency (Eizirik et al. 2009; Lucier and Weinstock 2022). Autoimmunity pathogenesis is difficult to reverse once the progression to type 1 diabetes begins. There has been much focus in the past decade on new approaches such as islet allografting (Pepper et al. 2018; Ridler 2016), gene therapy (Chellappan et al. 2018), stem cell therapy (Chen et al. 2020), and immunotherapies (Ni et al. 2019). However, these therapies are limited due to various reasons and have not been widely applied or promoted in clinical practice. On this note, a new paradigm targeting multiple pathogenic pathways is urgently needed for preventing or treating type 1 diabetes.

The autoimmune response triggering type 1 diabetes relies on the crosstalk between pancreatic beta cells and the immune system. Histology insulitis results obtained from experimental models of type 1 diabetes and type 1 diabetes patients demonstrate that macrophages and dendritic cells are the first cells that infiltrate into the islets (Nagy et al. 1989; Dahlén et al. 1998; Jörns et al. 2014; Walker et al. 1988; Hanenberg et al. 1989). Macrophage polarization is defined into two broad subsets: the classically activated M1 macrophages and alternatively activated M2 macrophages. The M1-type macrophages initiate insulitis and beta cell destruction whereas the M2-type macrophages suppress the immune response and are protective against type 1 diabetes (Espinoza-Jiménez et al. 2012). Therefore, the balance between M1 and M2 macrophage activation and polarization is crucial for disease progression.

The cellular metabolic adaptation to immune responses, immunometabolism is important in regulating the immune function of cells (Diskin and Palsson-McDermott 2018). Metabolic reprogramming is important in macrophage phenotype transition. Increased glycolysis and decreased oxidative phosphorylation are the main metabolic characteristics of M1-type macrophages (Torres et al. 2016). Alongside the enhanced glycolysis, tricarboxylic acid (TCA) cycle interruption is another significant characteristic of inflammatory macrophages, where metabolic intermediates accumulate and function as regulatory mediators of inflammatory responses (Ryan et al. 2019; Murphy and O'Neill 2018).

A derivative diverted from the TCA cycle, itaconate has recently become the focus of the immunometabolism field due to its anti-inflammatory properties that negatively regulate cytokine production and the inflammatory response (Mills et al. 2018). In accumulating studies, it was reported that itaconate is important in inflammatory-associated diseases via anti-inflammatory and antioxidant properties, such as acute lung injury (Xin et al. 2021; Li et al. 2020; Liu et al. 2021), autoimmune hepatitis (Yang et al. 2022), vitiligo (Xie et al. 2022), ischemia–reperfusion injury (Yi et al. 2020; Cordes et al. 2020), osteoclast-related diseases (Sun et al. 2019), cancer (Zhan et al. 2022), and renal fibrosis (Tian et al. 2020). The functions attributed to itaconate predominantly include aerobic glycolysis inhibition and transcription factor NF-E2-related factor 2 (Nrf2) activation (Mills et al. 2018; Liao et al. 2019). Notably, Nrf2 signaling activation contributed to ameliorating inflammation-mediated autoimmune disorders. Recently, it was proven that systemic activation of Nrf2 signaling delayed the onset of type 1 diabetes in spontaneous non-obese diabetic (NOD) mice (Yagishita et al. 2019), which suggested that Nrf2 is a potential target for preventing and treating type 1 diabetes. The cell-permeable itaconate derivative 4-octyl itaconate (OI) inhibited proinflammatory cytokine production in macrophages and reprogrammed them into an M2-like phenotype with a significant anti-inflammatory property (Lampropoulou et al. 2016; Tang et al. 2018). However, the effect of OI on inflammation in type 1 diabetes remains unclear. The critical effect of macrophage phenotype transition in innate immunity and the strong influence of OI on immune responses through Nrf2 activation prompted this investigation of the use of OI as a preventative, or even therapeutic, modality to deter type 1 diabetes progression in mouse models and exploration of the potential mechanism of macrophage phenotype reprogramming.

## Materials and methods

### Animals

Six-week-old male C57BL/6 mice (19–22 g) and 4-week-old female NOD mice (15–18 g) were obtained from the Model Animal Research Center (Nanjing, China). All mice were housed in a standard 12-h light–dark cycle and had free access to a normal chow diet with water ad libitum. The animal experiments were performed following national and institutional guidelines for the care and use of animals.

### Type 1 diabetes models and OI treatment

**Fig. 1:**
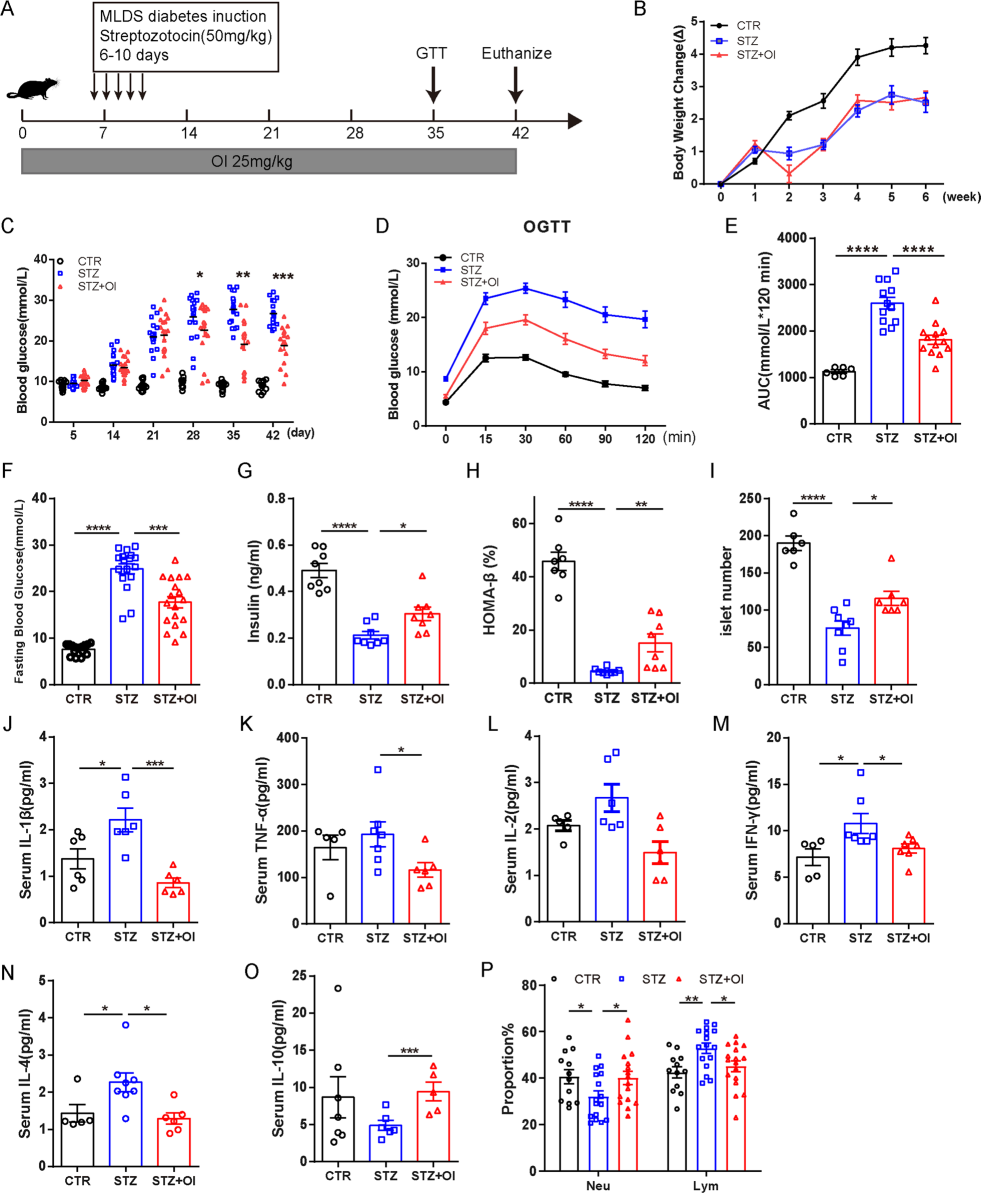
OI prevented glycemic deterioration, improved glucose metabolism and restored systemic inflammation in STZ-induced diabetes. **A** Schematic representation of the experimental protocol. **B** Mouse body weight change (*n* = 15–19 mice per group). **C** Random blood glucose measurements were performed throughout the study (*n* = 15–19 mice per group). **D** Week 5 OGTT results. **E** The AUC from the OGTT results in **D** (*n* = 6–13 mice per group). **F** Fasting blood glucose (*n* = 15–18 mice per group). **G** Fasting serum insulin concentration (*n* = 8 mice per group). **H** HOMA-β index (*n* = 7–8 mice per group). **I** Islet quantity from the mouse pancreas (*n* = 6–8 mice per group). **J**–**O** Serum cytokine concentrations (*n* = 5–8 mice per group). **P** The proportion of neutrophils (Neu) and lymphocyte (Lym) in mouse peripheral blood (*n* = 12–17 mice per group). All values are shown as the mean ± SEM. *p < 0.05, **p < 0.01, ***p < 0.001, and ****p < 0.0001. CTR, control

A male C57BL/6 mouse model of type 1 diabetes was established by multiple low doses of streptozotocin (STZ) (ESM Methods: Type 1 diabetes induction). In the prevention mouse model, OI (25 mg/kg, China) was dissolved in 2-hydroxypropyl-β-cyclodextrin (HBPCD, 45% wt/vol, China) in PBS and administered intraperitoneally 5 days before the first STZ dose and for 6 weeks (Fig. 1A). The OI and STZ injections were administered 3 h apart.

To confirm the effect of OI on diabetes prevention, the incidence of spontaneous autoimmune diabetes was assessed in female NOD mice. OI or HBPCD (control, CTR) was administered intraperitoneally once daily for up to 42 weeks (starting at the age of 4 weeks).

To investigate whether OI could reverse the hyperglycemia in type 1 diabetes, the STZ-induced diabetic mice received OI daily, which was initiated after hyperglycemia onset and continued for 12 weeks (Fig.  4A).

### Glucose tolerance test (GTT)

All C57BL/6 mice underwent the oral GTT (OGTT). Briefly, after overnight fasting with water ad libitum, mice were gavaged with a glucose solution in saline (1 g/kg using 20% dextrose solution).

The NOD mice underwent the intraperitoneal GTT (ipGTT). Briefly, after overnight fasting with water ad libitum, the mice were injected intraperitoneally with a glucose solution in saline (2 g/kg using 20% dextrose solution).

The blood glucose level from the tail vein was measured at baseline and at 15, 30, 60, and 120 min after glucose administration using a OneTouch Ultra blood glucose analyzer.

### Histological analysis

The mouse pancreases were paraffin-embedded and sectioned to 4 μm thickness. The sections were stained with Mayer’s hematoxylin to evaluate the mononuclear cell infiltration in the islets (ESM Methods: Insulitis score). For the TUNEL assay, the sections were stained with TUNEL assay (In Situ Cell Death Detection Kit, Roche). For immunofluorescence staining, the sections were assayed with DAPI as a counterstain. The primary antibodies are listed in Additional file 1: Table S1. Immunohistochemical analysis of inducible nitric oxide synthase (iNOS), F4/80, high-mobility group box 1 protein (HMGB1), and Nrf2 was performed as previously described (Yang et al. 2022). The relative content and positive cells in the islets were determined by ImageJ.

### Islet isolation and proteomics

The mouse pancreatic islets were isolated by collagenase type V digestion and purified using a density gradient as described previously (O'Dowd and Stocker 2020). The islets were washed in Hanks’ balanced salt solution (HBSS) and carefully hand-selected. The islet proteomics analysis in this study was performed by Jingjie PTM BioLabs (Hangzhou, China) (ESM Methods: Proteomics). To analyze the proteomics data, we used Venn diagrams and Mfuzz analysis to better visualize and identify the differentially expressed proteins (DFEs) between the three groups. To investigate the effect of OI on STZ-induced diabetes, we created a volcano plot and heat map of the pancreatic beta cell function in the STZ and OI groups. Moreover, we used the KEGG pathway database to annotate the DFEs pathways.

### Cell isolation and culture

Spleen and pancreatic lymph nodes (PLNs) were mechanically disassociated and passed through a 40-μm cell strainer to harvest single-cell suspensions. Splenocytes were acquired after removing erythrocytes with red blood cell (RBC) lysis buffer. Peritoneal macrophages were isolated from the control and STZ mice by i.p. injection of 2 ml 3% thioglycolate per mouse for 2 consecutive days. On day 4, the cells were harvested by peritoneal lavage of 10 ml DMEM containing 0.1% penicillin and streptomycin. After 10-min centrifugation at 400×*g*, the cells were washed with PBS once, resuspended in culture medium, and seeded in 6-well plates at 5 × 10^6^ cells/well. After adherence overnight in the incubator, non-adherent cells were removed by washing with PBS and changed to fresh medium before the intervention. Murine MIN6 beta cells were cultured in DMEM containing 10% FBS, 50 μM β-mercaptoethanol, and 0.1% penicillin and streptomycin. Peripheral blood mononuclear cells (PBMCs) were isolated from a 5 ml fresh whole blood sample using density gradient separation with Histopaque®-1077 (Sigma Aldrich). The cells were then plated in 12-well plates at 5 × 10^7^ cells/well. After adhering for 3 h in the incubator, the PBMCs that failed to adhere were removed by washing with cold PBS and changed to fresh medium before the intervention.

### Flow cytometry

The macrophage phenotype in the mouse PLNs was evaluated by flow cytometry using the following antibodies: CD16/32, CD45, CD11b, CD206 (all from BioLegend), and CD11c (Ebioscience). After 20-min blocking with CD16/32 at 4 °C, the surface markers were subsequently stained for 30 min at 4 °C. The cells were acquired on a BD LSRFortessa and analyzed by FlowJo. The data were first gated on live cells through viability dye, then further gated according to the required analysis.

### Glucose-stimulated insulin secretion (GSIS)

Peritoneal macrophages were obtained by peritoneal lavage. MIN6 cells were co-cultured with inflammatory macrophages or OI-pretreated macrophages for 24 h in transwell chambers (12-well plates) containing 0.4-μm pore filters (Fig. 7H). The MIN6 cells were washed with pre-warmed PBS, then pre-incubated in pre-warmed KRB containing 2.8 mM glucose for 0.5 h in a humidified atmosphere of 5% CO_2_ at 37 °C. Subsequently, the cells were incubated with pre-warmed KRB containing either 2.8 mM or 25 mM glucose for another 1 h. The collected supernatant was centrifuged at 400×*g* for 10 min to remove cells and debris. Then, the insulin content was measured using an ELISA kit (Ezassy, China) following the manufacturer’s instructions and normalized with the protein content.

### Participants

Nine non-diabetes participants aged > 18 years were included in the study. Ten type 1 diabetes patients were recruited from the diabetic ward of Sir Run Run Shaw Hospital of Zhejiang University. The patients comprised eight men and two women (mean age: 41.7 ± 15.2 years). Of these, 3 patients presented positive auto-immunity antibody. All patients were accompanied by ketosis or ketoacidosis. The fasting C-peptide in type 1 diabetes patients was below the lower limit of normal range. The clinical characteristic of the type 1 diabetes patients is summarized in Additional file 1: Table S3.

### Peripheral blood mononuclear cell (PBMC) stimulation

The PBMCs from non-diabetes participants and type 1 diabetes patients were pre-treated with 125 μM OI or NT for 2 h, followed by treatment with 200 ng/ml lipopolysaccharide (LPS), 125 μM OI + 200 ng/ml LPS (OI + LPS), or NT for another 24 h. The collected supernatant was centrifuged at 400×*g* for 10 min to remove cells and debris, and the cytokine concentrations were analyzed with an ELISA kit (Multisciences, China) following the manufacturer’s instructions.

### Statistical analysis

The data are expressed as the mean ± SEM for animal experiments or mean ± SD for cell experiments. The number of independent animal experiments is shown in the related figure legends, where* n* refers to the number of mice per group. All cell experiments were repeated at least three times. Statistical differences were analyzed using Student’s *t*-test and analysis of variance (ANOVA) (GraphPad Prism 9). A *p*-value < 0.05 indicated statistical significance. The difference in diabetes onset incidence between two groups of NOD mice was determined using the Mantel-Cox log-rank test.

## Results

### OI intervention improved glucose metabolism in mouse models of type 1 diabetes

In the prevention mouse model, OI was started 5 days before the first STZ dose and was administered for 6 weeks. Although the animals receiving OI exhibited similar body weight gain to the STZ-induced diabetic mice at the end of the study (Fig. 1B) OI prevented glycemic deterioration and improved the islet quantity of the animals that received STZ (Fig. 1C, I). During the OGTT, the OI mice exhibited significantly lower blood glucose levels than the STZ-alone mice at all time points (Fig. 1D, E). The improved glucose tolerance in the OI group was primarily attributed to the elevated plasma insulin level (Fig. 1G). Importantly, we did not detect adverse effects or liver and kidney function impairment after OI treatment (Additional file 1: Figures S1, S2). Besides, the spontaneous autoimmune diabetes model was used for further study. Five of 14 control NOD mice developed diabetes at 14–41 weeks of age. In contrast, none of the OI intervention mice were diabetic by the end of the study, which was significantly different (*p* = 0.012, Mantel-Cox log-rank test) (Fig. 3A). During the ipGTT, the NOD mice maintained on OI administration had better glucose homeostasis than the control NOD mice (Fig. 3B, C). The serum insulin level was significantly increased in the NOD mice with OI intervention compared to the control (Fig. 3D). To investigate whether OI could reverse the hyperglycemia in type 1 diabetes, OI treatment was initiated after hyperglycemia onset, where similar results of improved glucose homeostasis were observed in the treated type 1 diabetic mouse model (Fig. 4C, D).

### OI intervention restored the cytokine secretion profiles in STZ-induced diabetes

OI intervention significantly decreased the proinflammatory factors (IL-1β, TNF-α, IL-2, IL-4, IFN-γ) in the blood of STZ-induced diabetic mice but increased the levels of IL-10, which is an anti-inflammatory characteristic (Fig. 1J–O). In the treated type 1 diabetes mouse model, serum TNF-α, IL-12, and IFN-γ secretion were greatly reduced in the OI group, whereas CXCL1 and IL-10 production was restored (Additional file 1: Figure S7).

### OI intervention reduced pancreatic insulitis and restored beta cell function

**Fig. 2:**
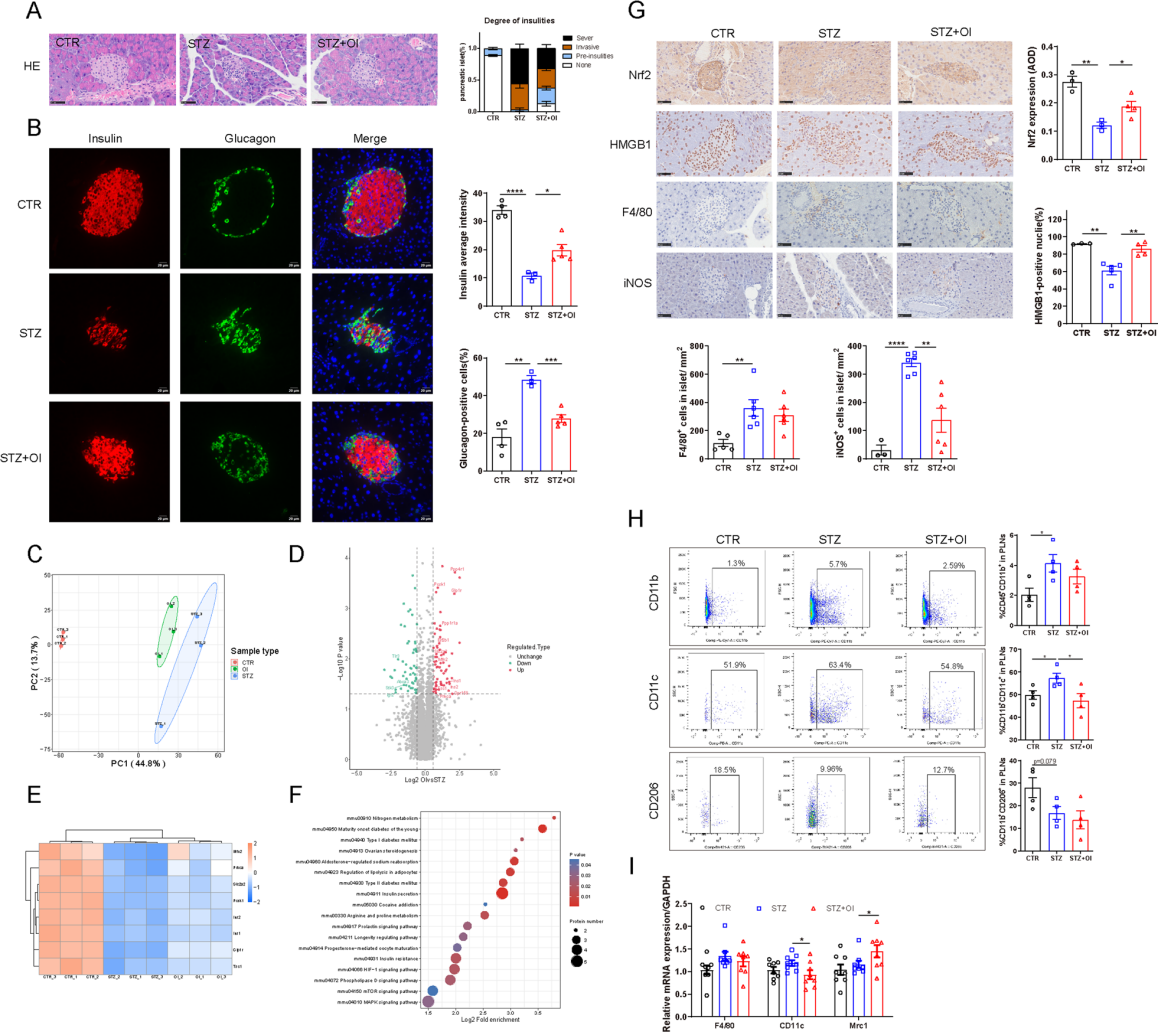
OI attenuated insulitis, improved beta cell function, and suppressed inflammatory macrophage infiltration in STZ-induced diabetes. **A** Representative images of pancreatic histology evaluated by H&E staining (*n* = 5–8 mice per group). The scoring criteria were as described in the ESM. **B** Representative images of pancreatic immunofluorescence staining for insulin (red), glucagon (green), and DAPI (blue). Insulin expression per islet was determined by analyzing fluorescence intensity with ImageJ. The percentage of glucagon-positive cells per islet was calculated in a bar graph (*n* = 3–5 mice per group). **C** Principal component analysis plot demonstrating the islet sample distribution in the control, STZ, and OI groups. The plot displays three distinct clusters (*n* = 3 mice per group). **D** Volcano plot depicting the 130 DFEs identified in the STZ and OI islets. **E** Heat map analysis illustrating the DFEs associated with islet beta cell function. The color change from blue to orange indicates low to high expression values. **F** List of Kyoto Encyclopedia of Genes and Genomes (KEGG) pathways significantly associated with the DFEs identified in the STZ and OI groups. **G** Immunohistochemistry analysis of Nrf2, HMGB1, F4/80, and iNOS expression in the mouse pancreas. Nrf2 expression was analyzed by ImageJ and presented as the Average Optical Density (AOD). The percentage of HMGB1-positive nuclei per islet was quantified in a bar graph. The F4/80- and iNOS-positive cells per islet area were calculated in a bar graph (*n* = 3–6 mice per group). **H** Flow cytometry assessment of the proportion of CD45^+^CD11b^+^, CD11b^+^CD11c^+^, and CD11b^+^CD206^+^ macrophages in PLNs along with representative dot plots (*n* = 4 mice per group). **I** *F4*/*80*, *Cd11c*, and *Mrc1* gene expression in splenocytes (*n* = 8 mice per group). All values are the mean ± SEM. *p < 0.05. All values are shown as the mean ± SEM. **p < 0.01, ***p < 0.001, and ****p < 0.0001

**Fig. 3:**
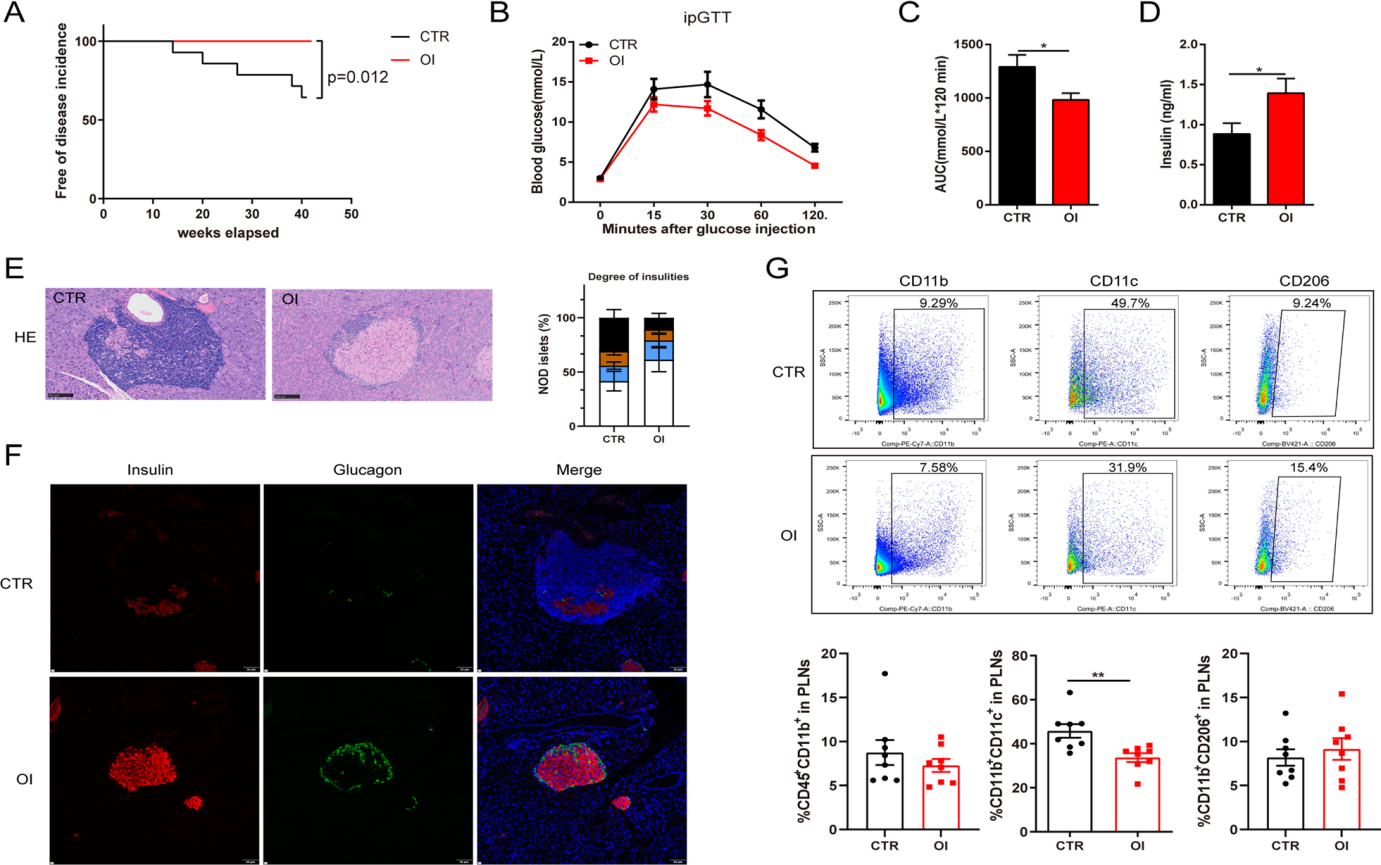
OI treatment reduced diabetes onset in spontaneous non-obese diabetic (NOD) mice.** A** Kaplan–Meier survival curves demonstrating the percentages of diabetes-free NOD mice (*n* = 14–15 mice per group). **B** Week 22 ipGTT results of NOD mice. **C** The AUC of the ipGTT results in B (*n* = 12–15 mice per group). **D** Serum insulin concentration of NOD mice (*n* = 12–13 mice per group). **E** Representative images of pancreatic histology evaluated by H&E staining (*n* = 11–12 mice per group). **F** Representative images of pancreatic immunofluorescence staining for insulin (red), glucagon (green), and DAPI (blue). **G** Flow cytometry assessment and the proportion of CD45^+^CD11b^+^, CD11b^+^CD11c^+^, and CD11b^+^CD206^+^ macrophages in PLNs and the representative dot plots (*n* = 8 mice per group). All values are shown as the mean ± SEM. *p < 0.05 and **p < 0.01. CTR, NOD-control

**Fig. 4:**
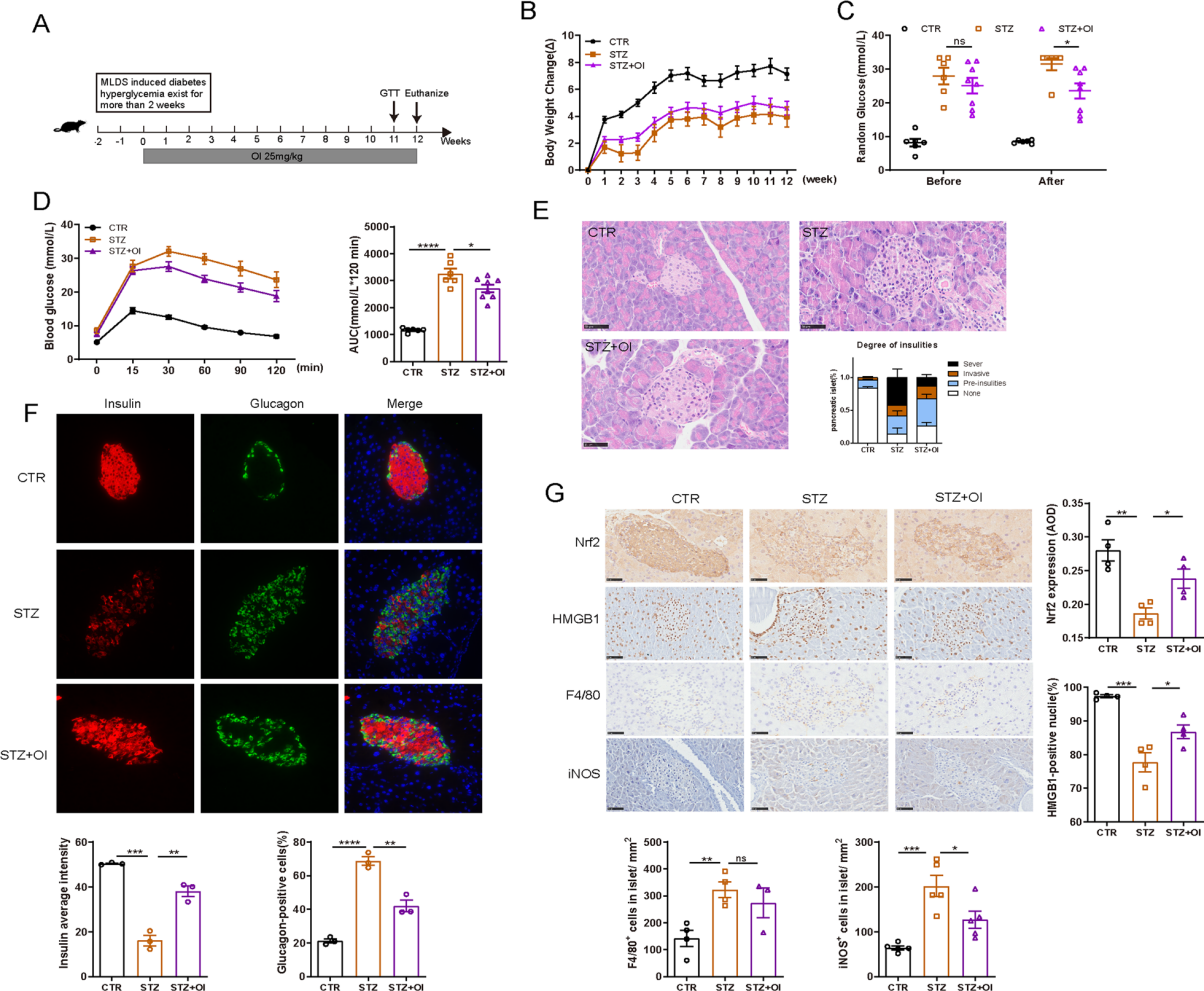
The therapeutic effect of OI on glucose homeostasis and inflammation in STZ-induced diabetes.** A** Schematic representation of the experimental protocol. C57BL/6 mice with STZ-induced diabetes received OI (25 mg/kg) daily after hyperglycemia onset and for 12 weeks. **B** Mouse body weight change (*n* = 6–9 mice per group). **C** Random blood glucose measurements were performed before the study and at the end of OI treatment. **D** Week 11 OGTT results. **E** Representative images of pancreatic histology evaluated by H&E staining (*n* = 5–7 mice per group). **F** Representative images of pancreatic immunofluorescence staining for insulin (red), glucagon (green), and DAPI (blue). Insulin expression per islet was determined by analyzing fluorescence intensity with ImageJ. The percentage of glucagon-positive cells per islet was calculated in a bar graph (*n* = 3 mice per group). **G** Immunohistochemistry analysis of Nrf2, HMGB1, F4/80, and iNOS expression in mouse pancreas. Nrf2 expression was analyzed by ImageJ and presented as the AOD. The percentage of HMGB1-positive nuclei per islet was quantified in a bar graph. The F4/80- and iNOS-positive cells per islet area were calculated in a bar graph (*n* = 3–5 mice per group). All values are shown as the mean ± SEM. **p < 0.01, ***p < 0.001, and ****p < 0.0001

Pancreatic H&E staining revealed that most islets in the STZ-induced diabetic mice exhibited severe insulitis and unclear margins, while the OI-treated group had a sharply reduced proportion of severe and invasive insulitis and restored islet structure (Fig. 2A). In NOD mouse pancreas, excessive inflammatory cells surrounded or invaded into islets, which OI significantly decreased (Fig. 3E). TUNEL assay demonstrated that the number of apoptotic cells was obviously increased in the STZ-induced diabetic mouse islets, which OI attenuated (Additional file 1: Figure S3). The islets in the STZ-induced diabetic animals contained very few insulin-positive cells and strong centralization of glucagon-staining cells (Fig. 2B). In marked contrast, OI administration increased the presence of functional insulin-positive beta cells and reduced glucagon-positive alpha cells similar to that in healthy islets. Principal component analysis of islets proteomics revealed distinct sample group clustering in the three groups (control, STZ, OI), with OI administration exerting less pronounced effects than STZ as compared with the control group (Fig. 2C). The DFEs in the OI vs. STZ groups are depicted in a volcano plot (Fig. 2D). The DFEs contained proteins relating to beta cell function, including MFN2, PRKCA, SLC2A2, PCSK1, INS1, INS2, GLP1R, and TSC1, which are summarized in a heat map (Fig. 2E). In the NOD mouse model, the insulin-positive cells were markedly destroyed by immune cells whereas they were restored by OI intervention (Fig. 3F). Similar findings of attenuated beta cell damage were also observed in the treated type 1 diabetic mouse model (Fig. 4E, F).

### OI intervention attenuated oxidative stress and suppressed proinflammatory macrophage infiltration

The protective effect of OI predominantly relied on its powerful activation of Nrf2, which is critical in oxidative stress responses (Mills et al. 2018). The results demonstrated greatly decreased Nrf2 expression after the mice received STZ, whereas OI intervention significantly reversed the islet Nrf2 content (Fig. 2G). In some of the infiltrated immune cells, HMGB1 translocated from the nucleus to the extracellular spaces, indicating HMGB1 active secretion (Zhang et al. 2009). Compared with diabetic mice, the islet cell nuclei of the OI-treated mice had a much higher proportion of HMGB1 expression, which implied that HMGB1 secretion was greatly inhibited (Fig. 2G). Although the STZ animals had much higher F4/80-positive cell infiltration in the islets compared to the controls, those cells were not significantly changed among the diabetes and OI groups. Surprisingly, the OI treatment mice had much lower numbers of iNOS-positive macrophages associated with the islets (Fig. 2G), which was also observed in the treated type 1 diabetes mouse model (Fig. 4G).

Phenotype analysis of PLN cells demonstrated that the STZ-induced diabetic mice had a greatly increased macrophage population (CD45^+^CD11b^+^) and percentage of CD11b^+^CD11c^+^ (proinflammatory M1) macrophages and a decreased proportion of CD11b^+^CD206^+^ (anti-inflammatory M2) macrophages as compared to the control. OI intervention obviously lowered the percentage of M1 macrophages in the PLNs of STZ-induced diabetic mice (Fig. 2H). Furthermore, OI significantly decreased *Cd11c* gene expression in the STZ-induced diabetic mouse splenocytes and increased *Mrc1* gene expression (Fig. 2I). In the NOD mouse model, the OI group had a markedly lower proportion of CD11b^+^CD11c^+^ (proinflammatory M1) macrophages in the PLNs as compared to the control (Fig. 3G).

### Effect of OI intervention in vitro on the peritoneal macrophage phenotype and mitogen-activated protein kinase (MAPK) pathway in STZ-induced diabetes

**Fig. 5:**
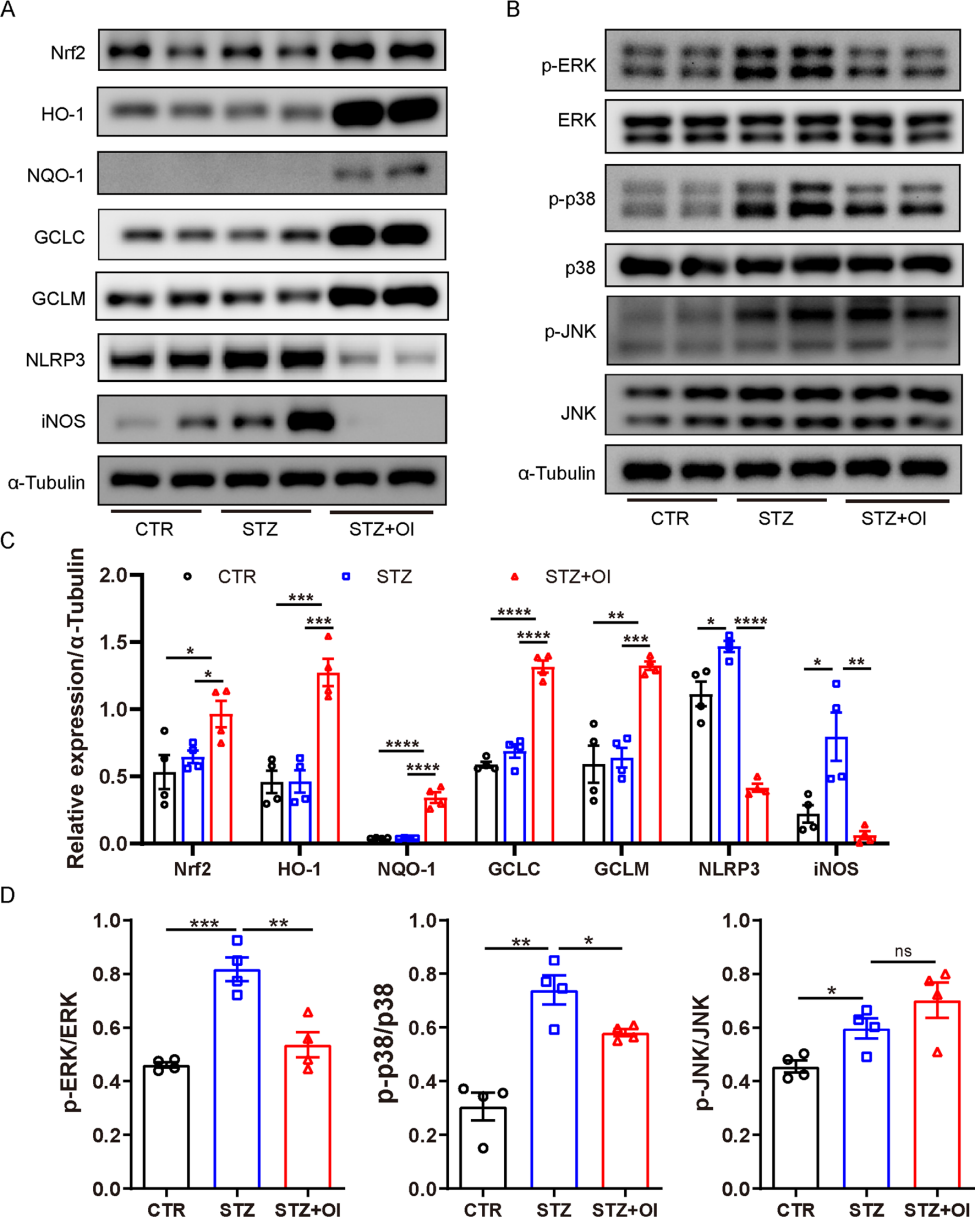
The effect of OI on peritoneal macrophages from STZ-induced diabetic mice. The peritoneal macrophages from healthy and diabetic mice were stimulated with OI or NT for 24 h. **A**,** C** Representative western blots and protein quantification of Nrf2, HO-1, NQO-1, GCLC, GCLM, NLRP3, and iNOS in peritoneal macrophage lysates (*n* = 4 mice per group). The internal control was α-tubulin. Band intensity was measured by ImageJ. The band intensities were quantified and normalized to α-tubulin. **B**, **D** Representative western blots and activity quantification of the phosphorylated and total levels of ERK, p38, and JNK in peritoneal macrophage lysates. The internal control was α-tubulin. The results are shown as the mean ± SD. *p < 0.05, **p < 0.01, ***p < 0.001, and ****p < 0.0001

Islets proteomics demonstrated that DFEs in the OI vs. STZ groups were significantly enriched in diabetes, insulin secretion, insulin resistance, and in the HIF1-α, mTOR, and MAPK signaling pathways (Fig. 2F). Furthermore, we observed MAPK pathway activity in the peritoneal macrophages from diabetic mice. Consistently, the MAPK pathway was greatly activated in macrophages, which presented as higher levels of phosphorylated (p)-ERK, p-p38, and p-JNK in macrophages from the diabetic animals than in the controls (Fig. 5B, D). Macrophages from the STZ-induced diabetic mice produced more NOD-like receptor thermal protein domain associated protein 3 (NLRP3) and iNOS than the cells from the control mice regardless of whether they were stimulated by LPS (Fig. 5A, C). As expected, OI intervention sharply elevated Nrf2 and its downstream antioxidative molecules (GCLC, GCLM, HO-1, NQO-1). Consistent with this, OI treatment attenuated p-ERK and p-p38 activation but did not affect p-JNK. Notably, OI decreased NLRP3 and iNOS production such that it was even lower than the baseline level in the control mice.

### Effect of OI intervention in vitro on antioxidative gene expression and macrophage polarization under LPS stimulation

**Fig. 6:**
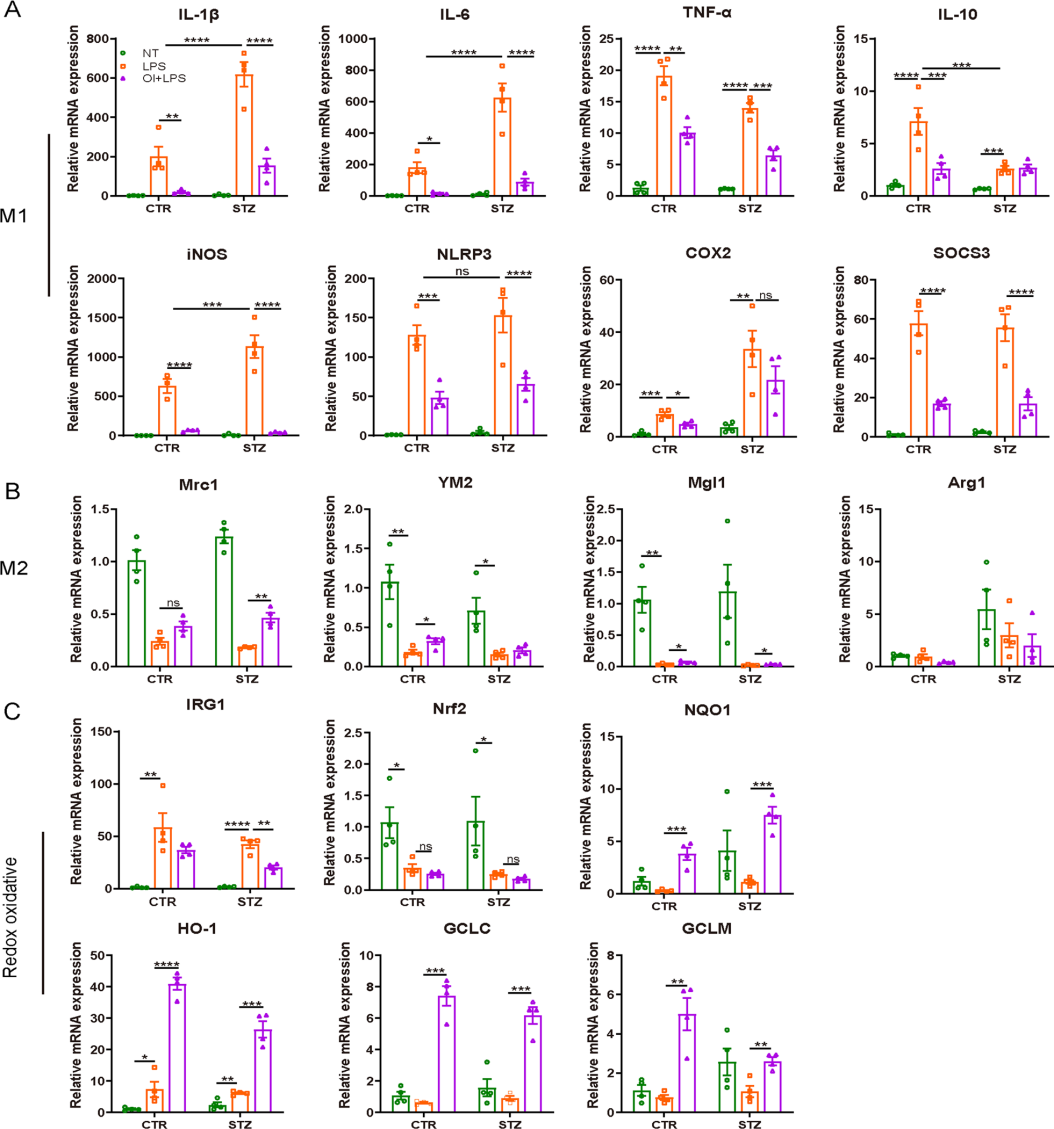
The effect of OI on oxidative stress gene expression and macrophage polarization. The peritoneal macrophages from healthy and diabetic mice were collected and were pretreated with 125 μM OI or NT for 2 h, followed by LPS, OI + LPS, or NT treatment. After 8-h treatment, total RNA was extracted and the expression of the M1-, M2-, and oxidative-related genes were measured by real-time PCR (*n* = 4 mice per group). The loading control was β-actin. **A** M1-related genes expression. **B** M2-related genes expression. **C** Oxidative-related genes expression. The results are shown as the mean ± SD. *p < 0.05, **p < 0.01, ***p < 0.001, and ****p < 0.0001

LPS induces monocyte/macrophage polarization to the M1 phenotype and was used in our study to activate macrophages. We challenged the diabetic and non-diabetic peritoneal macrophages with 100 ng/ml LPS and examined the expression of the M1/M2-related genes and antioxidative genes. LPS stimulation significantly upregulated *IRG1*, *HO-1*, *IL-1β*, *IL-6*, *TNF-α*, *iNOS*, *NLRP3, COX2, SOCS3,* and *IL-10* and downregulated *Nrf2, NQO1, GCLC, GCLM, Mrc1, YM1*, and *Mgl1* gene expression in the macrophage of control as seen in diabetic mice (Fig. 6). Notably, *IL-1β, IL-6, iNOS, NLRP3,* and *COX2* gene expression was much higher in the diabetic macrophages upon LPS stimulation, whereas *TNF-α* and *IL-10* gene expression was slightly and clearly decreased, respectively. The OI-pre-treated LPS-stimulated peritoneal macrophages had significantly increased *HO-1, NQO1, GCLC, GCLM, Mrc1, YM1*, and *Mgl1* gene expression and reduced *IL-1β, IL-6, TNF-α, iNOS, NLRP3*, and *SOCS3* gene expression, but OI did not affect Nrf2 transcription in the control or diabetic mice. Unexpectedly, OI treatment only attenuated *COX2* and *IL-10* gene expression in the healthy mouse peritoneal macrophages after LPS challenge.

### Peritoneal macrophages from the type 1 diabetic mice demonstrated an inflammatory state attenuated by OI treatment

**Fig. 7:**
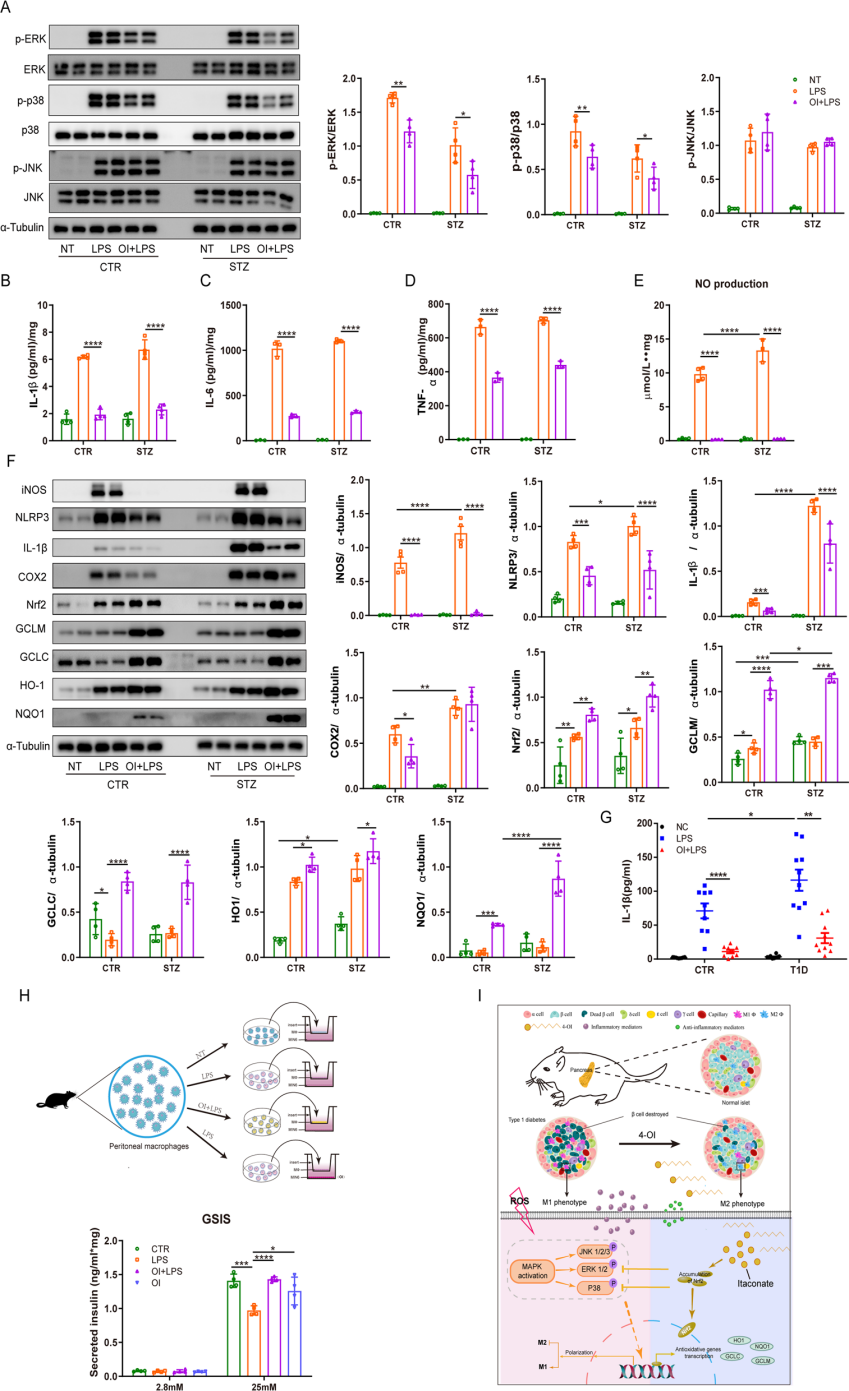
Peritoneal macrophages from diabetic mice demonstrated an inflammatory state that was attenuated by OI treatment. The peritoneal cells from healthy and diabetic mice were collected and were pretreated with 125 μM OI or NT for 2 h, followed by LPS, OI + LPS, or NT treatment (*n* = 4 mice per group). **A** Western blot analysis of the phosphorylated and total levels of ERK, p38, and JNK after 0.5-h treatment. **B–D** The cells and supernatant were collected after 24-h treatment. IL-1β, IL-6, and TNF-α levels were determined by ELISA and corrected with the protein content. **E** NO generation in the supernatant was measured by the Griess reaction. **F** Cell lysates underwent western blotting with Nrf2, HO-1, NQO-1, GCLC, GCLM, NLRP3, iNOS, IL-1β, COX2, and α-tubulin antibodies. The internal control was α-tubulin. **G** PBMCs collected from non-diabetes (CTR) and type 1 diabetic patients were adhered for 3 h, then pretreated with 125 μM  OI or NT for 2 h, followed by LPS, OI + LPS, or NT treatment. After 24-h treatment, IL-1β levels were in the supernatant (*n* = 9–10 per group). **H** Peritoneal macrophages were treated with or without LPS and OI for 8 h, then co-cultured with MIN6 cells for another 24 h. The supernatant was collected and GSIS was analyzed. The results are shown as the mean ± SD. *p < 0.05, **p < 0.01, ***p < 0.001, and ****p < 0.0001. **I** Schematic depicting the regulatory role of OI on macrophage polarization in protection against type 1 diabetes progression

We examined the proinflammatory cytokine concentrations in the supernatant of LPS-stimulated peritoneal macrophages with or without OI intervention. OI inhibited the release of proinflammatory factors such as IL-1β, IL-6, and TNF-α in the control and diabetic macrophages (Fig. 7B–D). Nitric oxide (NO) generation was more pronounced in the LPS-treated diabetic mice than in the controls, which was markedly suppressed to the basal level in both models (Fig. 7E). Similar to the above gene expression results, the diabetic macrophages contained higher IL-1β, iNOS, NLRP3, and COX2 protein levels compared with the control macrophages upon LPS stimulation, which were all attenuated by OI except for COX2 in diabetic mice (Fig. 7F).

After dissociating from KEAP1, Nrf2 accumulates and enters the nucleus to induce antioxidant protein expression (Mills et al. 2018). In this study, OI predominantly increased Nrf2, HO-1, NQO1, GCLC, and GCLM production in the LPS-treated control and diabetic macrophages (Fig. 7F). We also evaluated the MAPK pathway in macrophages after LPS stimulation. As expected, LPS treatment sharply increased the p-ERK, p-p38, and p-JNK levels in the macrophages. In both mouse models, OI reduced the p-ERK and p-p38 levels but not the p-JNK levels (Fig. 7A).

### OI intervention modulated IL-1β production in monocytes derived from type 1 diabetes patients in vitro

The above results prompted the investigation of whether OI exerts a similar effect on the human monocyte inflammatory response. The monocytes from type 1 diabetes patients exhibited higher IL-1β secretion potential in response to LPS than the monocytes from non-diabetic donors. Notably, the OI + LPS monocytes presented attenuated IL-1β secretion compared with LPS-only-treated cells from the non-diabetes or type 1 diabetes participants (Fig. 7G). Therefore, OI exhibited an anti-inflammatory impact on the monocytes of type 1 diabetes patients.

### Activated macrophages induced beta cell dysfunction and were restored by OI treatment

We designed a co-culture system to clarify the protective effect of OI on beta cell function via regulating macrophage polarization. Co-culture with LPS-stimulated activated macrophages greatly impaired the insulin release of MIN6 cells upon glucose stimulation as compared to co-culture with unstimulated macrophages. Surprisingly, macrophages pre-treated with OI before LPS stimulation significantly restored the impaired GSIS of the MIN6 cells. Moreover, OI pre-treatment before co-culture with activated macrophages improved glucose-stimulated insulin secretion in the MIN6 cells (Fig. 7H).

## Discussion

In this study, we demonstrated that the cell-permeable itaconate derivative OI ameliorated glycemic deterioration, improved impaired insulin secretion, and reduced insulitis in STZ-induced diabetic mice and delayed autoimmune diabetes onset in NOD mice. Notably, OI suppressed systemic inflammatory cytokine levels and attenuated pancreatic beta cell damage by inhibiting M1 macrophage activation through the Nrf2–MAPK pathway. Moreover, the macrophages from diabetic mice and monocytes from type 1 diabetes patients presented an inflammatory status that was attenuated by OI treatment.

It has been suggested in an increasing number of studies that OI is a novel and promising anti-inflammatory metabolite in restricting immunopathology and inflammation. However, its role in type 1 diabetes disease progression is unclear. Accordingly, we used multiple low-dose STZ-induced diabetes and spontaneous autoimmune diabetes to evaluate the effect of OI on type 1 diabetes prevention and treatment and explore the potential mechanism of macrophage phenotype reprogramming. OI significantly restored pancreatic beta cell function, which was consistent with improved glucose metabolism and less immune cell infiltration within or near the islets as compared to the diabetic mice. These results were also reported in a previous study, where the Nrf2 activator dimethyl fumarate protected beta cells against oxidative stress and reduced the incidence of spontaneous autoimmune diabetes in female NOD mice by attenuating insulitis and the level of circulating proinflammatory cytokines (Li et al. 2021). Cytokines are crucial in orchestrating complex multicellular interactions between pancreatic beta cells and immune cells in type 1 diabetes development. Proinflammatory cytokines are thought to lead to type 1 diabetes onset and progression. By contrast, cytokines that induce regulatory functions are thought to generate feedback regulation of diverse immune responses and protect against beta cell destruction (Lu et al. 2020). Our data demonstrated that the serum proinflammatory cytokines IL-1β, TNF-α, IL-2, IFN-γ, and IL-4 and the anti-inflammatory cytokine IL-10 were dysregulated in diabetes and improved by OI intervention.

The effectiveness of OI in ameliorating the glucose metabolism of type 1 diabetes was related to reduced inflammation in the pancreas. Despite the adaptive immune system playing a central role in the inflammation associated with type 1 diabetes, pancreatic beta cell dysfunction and activated M1 macrophage infiltration are early features in the disease pathogenesis (Burg and Tse 2018). Activated macrophages produce a series of inflammatory cytokines, such as IL-1β, TNF-α, and IFN-γ, which are critical in beta cell dysfunction and apoptosis (Burg and Tse 2018; Delmastro and Piganelli 2011). Histological analysis of pancreatic sections from both patients with type 1 diabetes and mouse models of autoimmune diabetes revealed an influx of recruited macrophages to the islets (Hänninen et al. 1992; Itoh et al. 1993; Jansen et al. 1993; Kolb-Bachofen et al. 1992; Roep et al. 1992). Zhang et al. reported that more inflammatory macrophages infiltrated into the islet cells in STZ-induced diabetic mice and that enhancing M1 macrophage activation further exacerbated pancreas injury (Zhang et al. 2020). Inhibiting macrophage infiltration into the islet cells or restricting macrophage M1 polarization in diabetic mice would be helpful for maintaining pancreas function and preventing type 1 diabetes progression. Notably, our results provided evidence supporting the hypothesis that the primary site of action of OI protection against diabetes might be the infiltrating macrophages and the subsequent cascade of local inflammatory events. Indeed, the macrophage infiltration was not significantly different among the diabetic and OI groups in our study. Interestingly, the OI mice had lower numbers of islet-associated M1 macrophages and a lower proportion of M1 macrophages in different peripheral compartments, which indicates a possible direct effect of OI on macrophage polarization.

However, the role of OI in macrophage phenotype reprogramming in diabetic mice remains complex. Metabolic alterations followed by diabetic progression are also associated with macrophage polarization. Hyperglycemia in diabetes induces epigenetic changes that reprogram the macrophage phenotype and modify the subsequent cellular response upon stimulus (Ratter et al. 2018). Macrophages derived from diabetic mice presented more abundant NLRP3 and iNOS protein expression and higher inflammatory cytokine secretion and NO production after LPS stimulation compared with cells from non-diabetic mice (Davanso et al. 2021). Moreover, BMDM from alloxan-induced diabetes impaired signaling pathways, which involved alterations at both PI3K–Akt and MAPK levels (Galvao Tessaro et al. 2020). Accompanying this, both BMDM and peritoneal macrophages from diabetic mice demonstrated dysregulated cytokine secretion profiles (Galvao Tessaro et al. 2020). Consistent with these literature data, our results supported the idea that OI reduced NLRP3 and iNOS expression and decreased NO production and proinflammatory cytokine release in macrophages upon LPS administration, which implied that OI significantly restricted macrophage M1 polarization.

Although the anti-inflammatory and antioxidative effects of OI have been reported, its distinct role in regulating the MAPK signaling response to inflammation is unknown. Mounting evidence suggested that the MAPK family is critical for regulating proinflammatory cytokines and mediators. MAPK pathway activation phosphorylates various downstream targets to induce inflammation mediator production in macrophages. It was proven in several studies that Nrf2 exerted anti-inflammatory and antioxidative functions by regulating MAPK pathway activity. It was suggested that Nrf2 activation mitigated oxidative stress and inflammation in mesangial cells caused by high glucose through inhibiting MAPK signaling (Yao et al. 2022). Moreover, sulforaphane exerted anti-neuroinflammatory effects on LPS-activated microglia through Nrf2–HO-1 pathway activation and JNK–AP-1–NF-κB pathway inhibition (Subedi et al. 2019). It is widely recognized that OI inhibits LPS-induced proinflammatory cytokine secretion, NF-κB activation, and oxidative stress. Here, our data indicate another pathway behind OI regulation of the inflammatory response in macrophages by attenuating p-ERK and p-p38 MAPK activation in diabetic macrophages and response to LPS stimulation. This result was consistent with our proteomics analysis results, where MAPK pathway-related proteins were greatly enriched in the DFEs between the OI intervention and diabetic mouse islets.

It has been reported in several studies that monocytes from new-onset type 1 diabetes patients have increased IL-1β basal levels (Meyers et al. 2010) and a more pronounced response to LPS stimulation in vitro (Davanso et al. 2021). Similarly, during the in vitro treatment of LPS-stimulated PBMCs isolated from type 1 diabetes patients, we observed substantially increased IL-1β secretion compared to the non-diabetes population. Our findings supported the idea that type 1 diabetes patients present a basal inflammatory status. Surprisingly, OI treatment of the monocytes attenuated IL-1β production compared with LPS-only stimulation in both non-diabetes and type 1 diabetes participants. The anti-inflammatory effect was consistent with that reported in a previous study, where OI activated Nrf2 signaling to inhibit proinflammatory cytokine secretion in the PBMCs of systemic lupus erythematosus patients (Tang et al. 2018).

As mentioned above, pancreatic beta cell dysfunction is closely related to activated M1 macrophage infiltration in type 1 diabetes pathogenesis. Apart from presenting antigens to autoreactive T cells in the initiation and effector phases of type 1 diabetes, activated M1 macrophages produce proinflammatory cytokines and NO to induce beta cell dysfunction and apoptosis (Burg and Tse 2018). In the present study, we regulated macrophage polarization in a co-culture system to investigate the protective effect of OI on beta cell function. The activated macrophages caused beta cell dysfunction whereas OI treatment significantly restored beta cell function. In addition, not only did OI regulate macrophage polarization, it might have distinctly improved beta cell function, which warrants further investigation.

Overall, our results demonstrated that OI was a potent activator of the Nrf2-mediated antioxidative response in macrophages and subsequently inhibited M1 polarization through the MAPK pathway. These actions might be crucial to the crosstalk between innate immunity and beta cell function and establish the basis for the emerging therapeutic implications of OI in type 1 diabetes progression.

## Conclusions

We provided the first evidence to date that the OI can impede islet inflammation progression and improve glucose metabolism by regulating macrophage phenotype reprogramming in mouse models of type 1 diabetes. Furthermore, OI significantly inhibited MAPK activation in macrophages to alleviate the inflammatory response of the pancreas, eventually improving beta cell dysfunction. The findings indicated that elevating endogenous itaconate levels might attenuate systemic inflammation, which provided potential new insight into a feasible adjuvant therapy for preventing and treating type 1 diabetes. Although this tentative investigation provided an important basic finding toward the development of a new drug class target for preventing and treating type 1 diabetes, significant hurdles remain and in-depth studies are warranted, as are further supporting studies in other models of diabetes.

## Supplementary Information

**Additional file 1.** The experimental protocols of prevention model and treatment model were presented in Fig. 1A and Fig. 4A, separately.

## Data Availability

The datasets generated during and/or analyzed during the current study are available from the corresponding author on reasonable request.

## Abbreviations

AOD: Average Optical Density
COX2: Cytochrome c oxidase subunit II
CTR: Control group that received vehicle alone
DFEs: Differentially expressed proteins
GCLC: γ-Glutamyl cysteine ligase catalytic subunit
GCLM: γ-Glutamyl cysteine ligase modifier subunit
GSIS: Glucose-stimulated insulin secretion
HBPCD: (2-Hydroxypropyl)-β-cyclodextrin
HO1: Heme oxygenase-1
ipGTT: Intraperitoneal glucose tolerance test
iNOS: Inducible nitric oxide synthase
KEGGs: Kyoto Encyclopedia of Genes and Genomes
LPS: Lipopolysaccharide
MAPK: Mitoge-activated protein kinase
NOD: Non-obese diabetic
NLRP3: NOD-like receptor thermal protein domain associated protein 3
NO: Nitrite oxide
Nrf2: NF-E2-related factor 2
NT: Cells which treated with vehicle
NQO1: NAD(P)H:quinone oxidoreductase 1
OGTT: Oral glucose tolerance test
OI: 4-Octyl itaconate
OI + LPS: Cells which treated with OI and LPS
PBMCs: Peripheral blood mononuclear cells
PCNA: Proliferating cell nuclear antigen
STZ: Streptozotocin
STZ + OI: STZ-induced diabetic mice that were treated with OI
TCA: Tricarboxylic acid
