# Supplementary material for: 4-Octyl itaconate attenuates glycemic deterioration by regulating macrophage polarization in mouse models of type 1 diabetes
Source: Mol Med. 2023 Mar 14;29:31. doi: 10.1186/s10020-023-00626-5 (PMC10015936; doi:10.1186/s10020-023-00626-5)
Supplement: Supplementary file 1 — Additional file 1. The experimental protocols of prevention model and treatment model were presented in Fig. 1A and Fig. 4A, separately. [file 10020_2023_626_MOESM1_ESM.docx]

**Electronic Supplementary Material**

**ESM method**

**Type 1 diabetes induction**

Animals were acclimatized for 1 week prior to being used in the experiment. Type 1 diabetic mouse model was induced in 8-week-old male C57BL/6 mice using multiple low doses of streptozotocin (STZ) that were administered intraperitoneally daily for 5 consecutive days. STZ (50 mg/kg bw, Shanghai, China) was dissolved in cold 0.1 M citrate buffer (pH = 4.2) just prior to administration.

The mice were monitored for the development of diabetes by weekly measurement of blood glucose levels and body weight change. Blood was collected by tail snipping and the glucose levels were measured using a OneTouch Ultra blood glucose analyzer. Animals were considered hyperglycemic if their random blood glucose level was >16.7 mmol/L.

**Peripheral blood cells and serum analysis**

Blood was obtained by cardiac puncture. Whole blood (200 µl) was analyzed by a scil Vet abc hemcounter. The remaining samples were centrifuged at 3000 rpm for 10 min, and the serum was frozen at −80°C, then analyzed for pro- and anti-inflammatory cytokines with a Luminex fluorescent bead array system. Alanine transaminase (ALT), aspartate transaminase (AST), alkaline phosphatase (ALP), lactate dehydrogenase (LDH), creatine kinase (CK), triglycerides (TG), total cholesterol (TC), HDL-c and LDL-c were measured using an automated biochemical instrument.

**Insulitis score**

Insulitis scoring was performed by examining at least 20 islets per pancreas and grading them as follows: 1) no insulitis; 2) peri-insulitis (leukocytes in the islet periphery); 3) invasive insulitis (25–50% coverage of the islet); and 4) severe insulitis (>50% infiltration). Results are expressed as the percentage of graded islets out of the total number of islets.

**Proteomics**

The proteomics analysis in this study was performed by Jingjie PTM BioLabs (Hangzhou, China). The primary experimental procedures for 4D label-free proteomics analysis included protein extraction, tryptic digestion, LC-MS/MS analysis, and bioinformatics analysis. The fold change cutoff was set when proteins with quantitative ratios >1.5 or <1:1.5 and p < 0.05 were deemed significant.

**Immunoblot analysis**

Western blotting analysis was performed as described previously(1). The primary antibodies are listed in Supplementary Table S1. After incubation with horseradish peroxidase-conjugated anti-mouse or anti-rabbit secondary antibodies, the bands were visualized by a gel imaging system (Bio-Rad) and quantified by densitometric analysis with ImageJ. The control reference was α-tubulin. Band intensity was measured by ImageJ. The band intensities were quantified and normalized to α-tubulin or total levels of ERK, p38, and JNK separately.

**Total RNA isolation and real-time PCR**

Total RNA extracted from splenocytes and peritoneal macrophages was reverse-transcribed into cDNA using an Evo M-MLV RT Premix kit (Accurate Biotechnology, Changsha, China). Real-time PCR was performed using a SYBR Green Premix Pro Taq HS qPCR kit (Accurate Biotechnology) and a QuantStudio 6 Flex Real-Time PCR System (Applied Biosystems). Gene expression levels were normalized to actin or *GAPDH* and calculated by the comparative threshold cycle (ΔΔCT) method. The primer sequences are summarized in *Supplementary Table S2*.

**Proinflammatory cytokine detection**

The IL-1-β, IL-6, and TNF-α concentrations in the cell-free culture supernatants were measured using ELISA kits (Multisciences) according to the manufacturer’s protocol and corrected with the protein content. Four replicates were performed for each treatment.

**NO generation assays**

The NO level in the culture supernatant was measured by a NO Assay kit (Beyotime, Beijing, China). Briefly, 50 μl cell-free culture supernatant followed by an equal amount of Griess reagent I and II were added to one well of a 96-well plate. The absorbance of each well was measured at 540 nm with a microplate reader (BioTek, Bad Friedrichshall, Germany) and the actual concentration was calculated according to the manufacturer’s instructions and corrected with the protein content.

**ESM figures**

**Supplementary Fig. S1** The effect of OI on the organ index in STZ-induced diabetes (*n* = 5–10 mice per group). (**A**) Pancreas index. (**B**) Liver index. (**C**) Thymus index. (**D**) Spleen index. All values represent the mean ± SEM. **p* < 0.05.
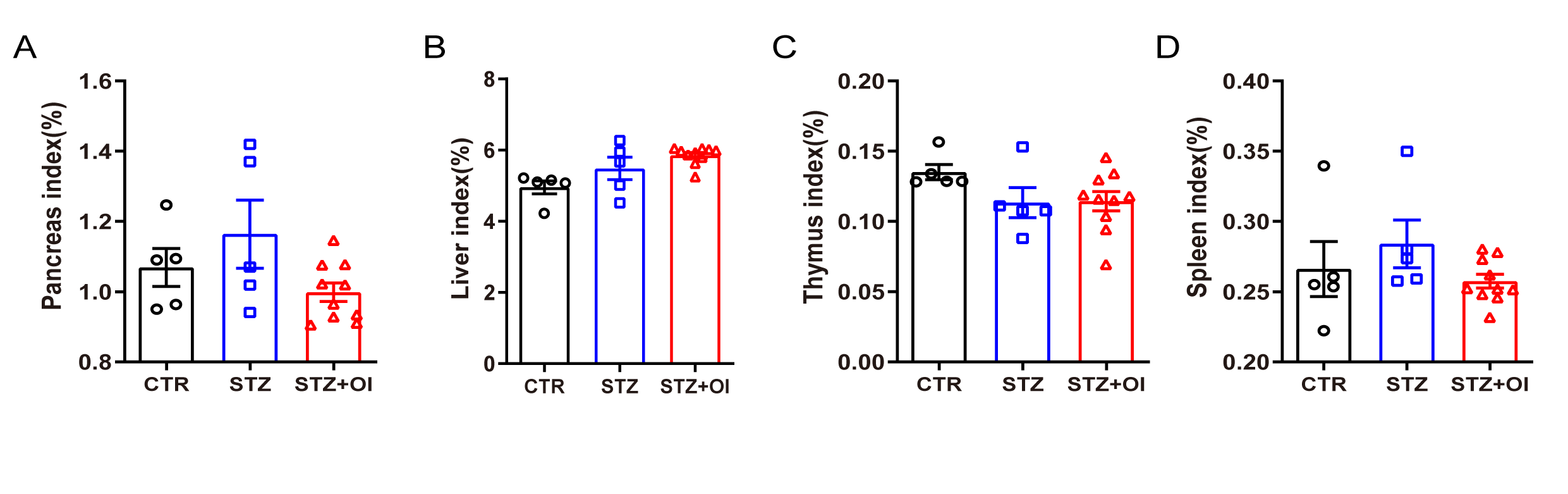


**Supplementary Fig. S2** The effect of OI on biochemical indicators in STZ-induced diabetes (*n* = 6–10 mice per group). (**A**) Serum liver function indicators. (**B**) Serum CK and LDH. (**C**) Serum lipid profiles. (**D**) Serum albumin. All values represent the mean ± SEM. ***p* < 0.01, ****p* < 0.001 and *****p* < 0.0001.


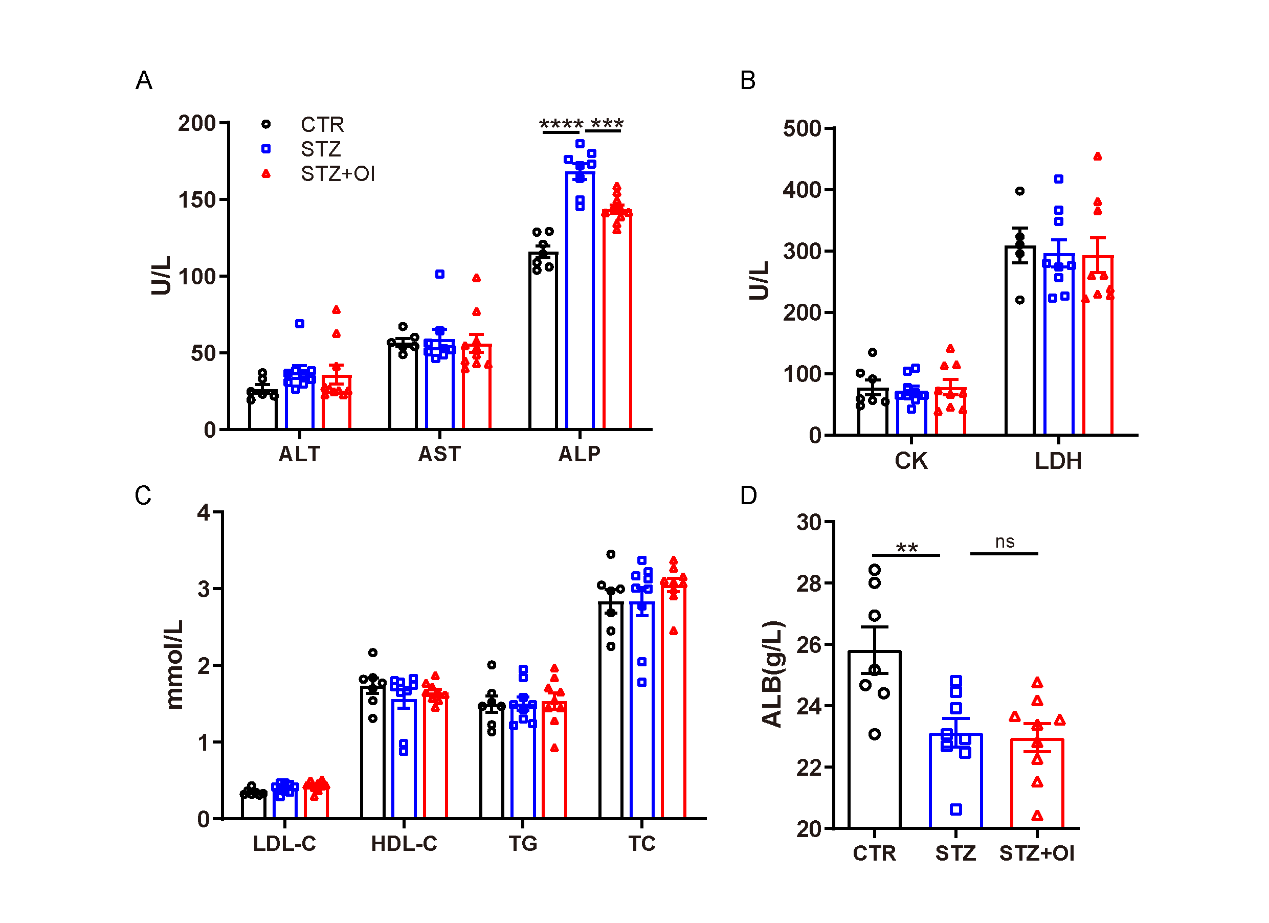


**Supplementary Fig. S3** The effect of OI on apoptotic and proliferative levels in islets from STZ-induced diabetic mice. The islet apoptotic level was evaluated by the amount of TUNEL-positive cell nuclei per islet area (*n* = 7-8 mice per group); The islet proliferative level was evaluated by the amount of PCNA-positive cell nuclei per islet area (*n* = 9-11 mice per group). All values represent the mean ± SEM. **p* < 0.05, ***p* < 0.01.


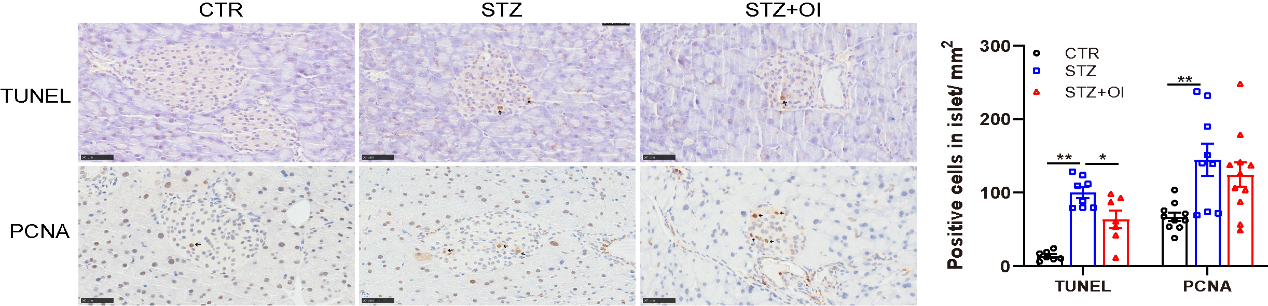


**Supplementary Fig. S4** Proteomics analysis of islets isolated from STZ-induced diabetes mice with OI prevention. Eight-week-old male C57BL/6J mice were treated with vehicle or OI 5 days before the first STZ dose and for 6 weeks. Multiple low-dose STZ injections (50 mg/kg) were administered for 5 consecutive days. (**A**) Venn diagram depicting the overlap between the DFEs identified from the CTR, STZ, and OI islets (*n* = 3 mice per group). (**B**) Histogram depicting the DFEs in the CTR, STZ, and OI islets. (**C**) Mfuzz analysis results of the six clusters. Red and purple represent the proteins with high membership values and more alignment with trend while yellow and green represent the genes with low membership values and less alignment with trend.


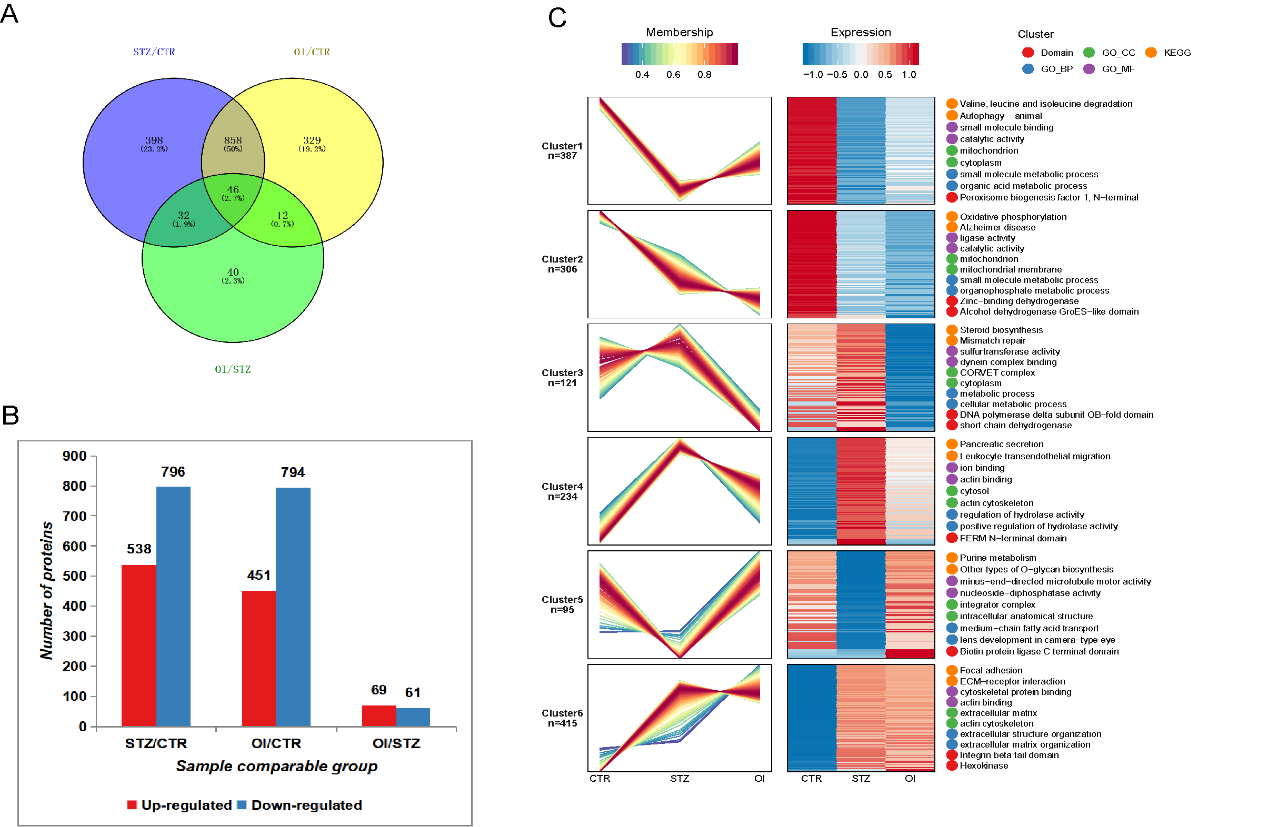


**Supplementary Fig. S5** The effect of OI on liver and kidney index in the treated type 1 diabetes mouse model (*n* = 5–7 mice per group). (**A**) Liver index. (**B**) Kidney index. All values represent the mean ± SEM. **p* < 0.05, ****p* < 0.001 and *****p* < 0.0001.


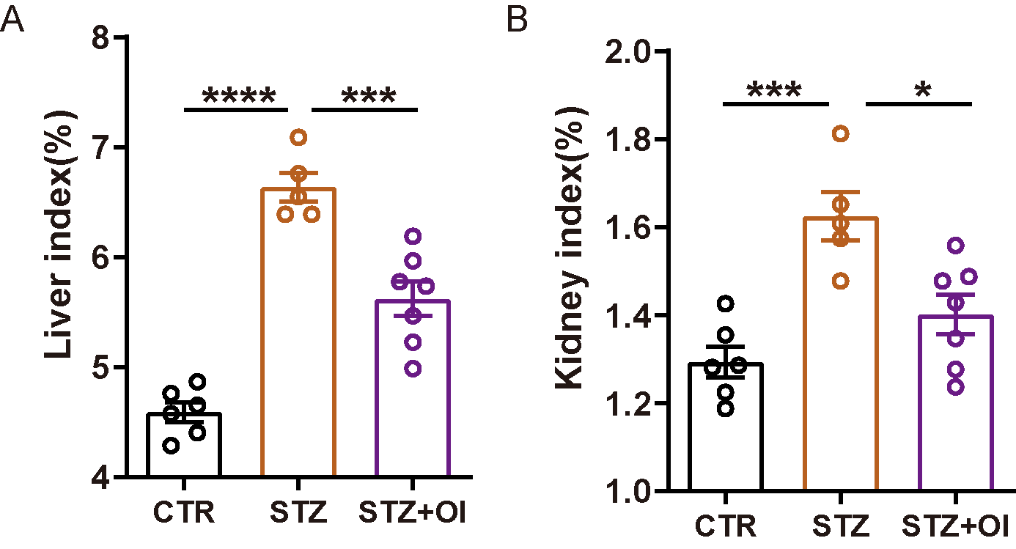


**Supplementary Fig. S6** The effect of OI on liver function and lipid profiles in the treated type 1 diabetes mouse model (*n* = 5-7 mice per group). (**A**) Serum liver function indicators. (**B**) Serum lipid profiles. All values represent the mean ± SEM. **p* < 0.05, ***p* < 0.01 and *****p* < 0.0001.


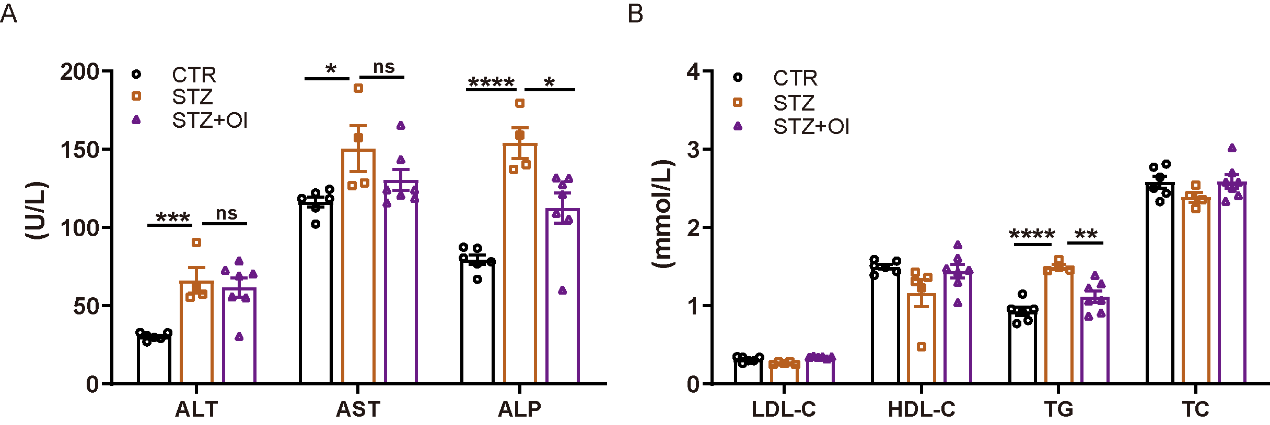


**Supplementary Fig. S7** Serum cytokine concentration in the treated type 1 diabetes mouse mode (*n* = 3–6 mice per group). (**A**) Serum IL-12 level. (**B**) Serum TNF-α level. (**C**) Serum IFN-γ level. (**D**) Serum CXCL1 level. (**E**) Serum IL-10 level. All values represent the mean ± SEM. **p* < 0.05 and ***p* < 0.01.


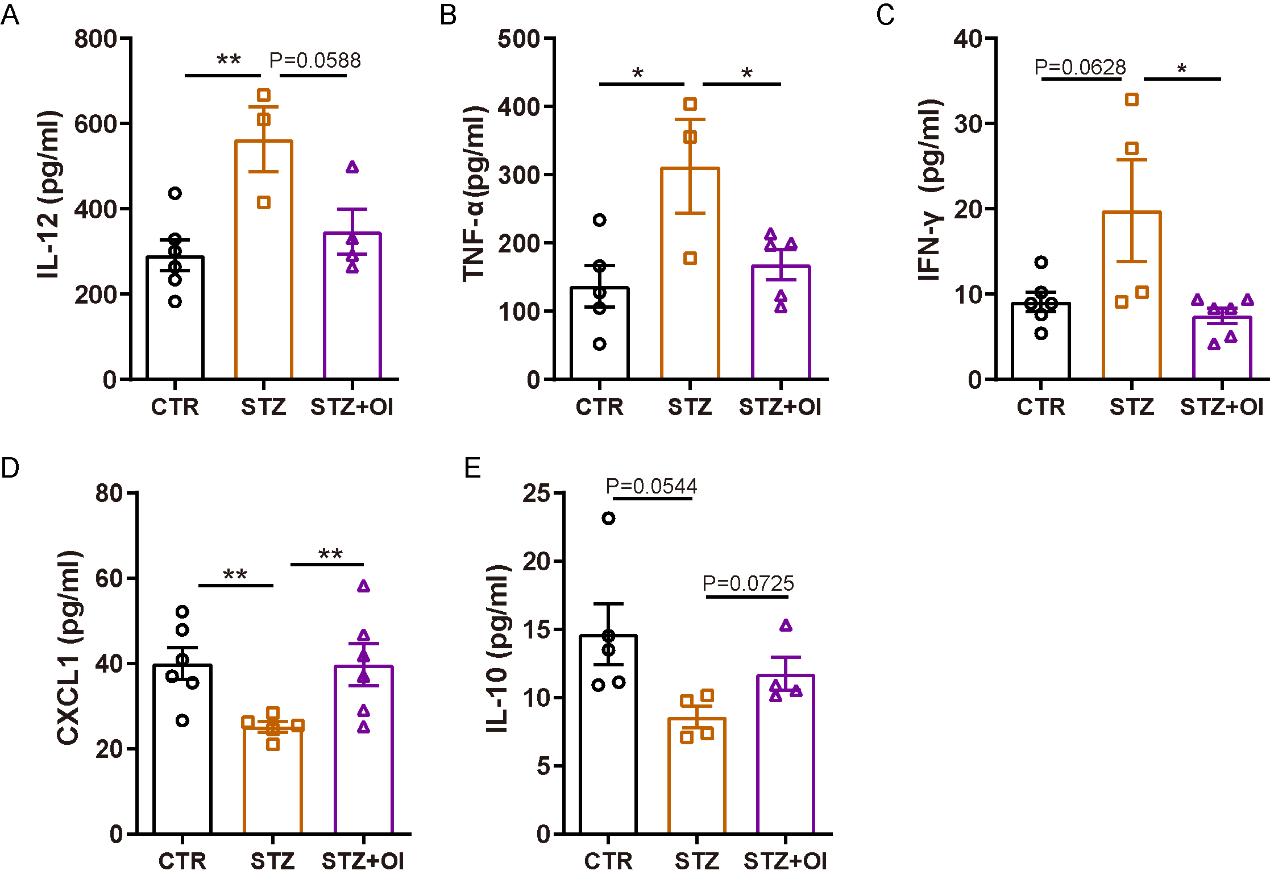


**ESM tables**

**Supplementary Table S1** The primary antibodies used in the study

| Primary antibody | Dilution ratio | Function | Supplier |
| --- | --- | --- | --- |
| p-ERK | 1:1000 | WB | Cell Signaling Technology |
| ERK | 1:1000 | WB | Cell Signaling Technology |
| p-P38 | 1:1000 | WB | Cell Signaling Technology |
| P38 | 1:1000 | WB | Cell Signaling Technology |
| p-JNK | 1:1000 | WB | Cell Signaling Technology |
| JNK | 1:1000 | WB | Cell Signaling Technology |
| Nrf2 | 1:1000 | WB | Abclonal |
| GCLC | 1:20000 | WB | Abcam |
| GCLM | 1:1000 | WB | Abcam |
| NQO1 | 1:10000 | WB | Abcam |
| HO1 | 1:2000 | WB | Abclonal |
| NLRP3 | 1:1000 | WB | Adipogen |
| iNOS | 1:1000 | WB, IHC | Abcam |
| Cox2 | 1:1000 | WB | Cell Signaling Technology |
| IL-1β | 1:1000 | WB | Abcam |
| Insulin | 1:400 | IF | Proteintech |
| Glucagon | 1:300 | IF | Proteintech |
| F4/80 | 1:200 | IHC | Proteintech |
| HMGB1 | 1:350 | IHC | Abcam |
| Nrf2 | 1:200 | IHC | Proteintech |
| PCNA | 1:200 | IHC | Proteintech |
| α-tubulin | 1:1000 | WB | Cell Signaling Technology |

**Supplementary Table S2** The primer sequences used in the study.

| Genes | Forward (5’-3’) | Reverse (5’-3’) |
| --- | --- | --- |
| *β-actin* | AGGCCAACCGTGAAAAGATG | AGAGCATAGCCCTCGTAGATGG |
| *GAPDH* | CATCACTGCCACCCAGAAGACTG | ATGCCAGTGAGCTTCCCGTTCAG |
| *IL-β* | TCGCAGCAGCACATCAACAAGAG | TGCTCATGTCCTCATCCTGGAAGG |
| *TNF-α* | GCGACGTGGAACTGGCAGAAG | GCCACAAGCAGGAATGAGAAGAGG |
| *iNOS* | GAGCGCTCTAGTGAAGCAAAG | CTTGCAAGTGAAATCCGATGTGG |
| *IL-6* | CAGAGGATACCACTCCCAAC | CAATCAGAATTGCCATTGCAC |
| *Cox2* | TGGTGCCTGGTCTGATGATG | GTGGTAACCGCTCAGGTGTTG |
| *NLRP3* | CAAGGCTGCTATCTGGAGGAA | TGCAACGGACACTCGTCATC |
| *SOCS3* | GACCAAGAACCTACGCATCCAGTG | GCACCAGCTTGAGTACACAGTCG |
| *Mrc1* | CCTATGAAAATTGGGCTTACGG | CTGACAAATCCAGTTGTTGAGG |
| *KLF4* | ACCTCCTGGACCTAGACTTTAT | GAAGACGAGGATGAAGCTGAC |
| *Mgl1* | ATGGGTGGATGGGACCGACTTT | GGAAGGTTCTCTGGCAGACATC |
| *IL-10* | GCCAAGCCTTATCGGAAATG | CACCCAGGGAATTCAAATGC |
| *YM1* | AGAAGCTCTCCAGAAGCAATCC | CATCAGCTGGTAGGAAGATCCCAG |
| *Nrf2* | GAAGCTCAGCTCGCATTGATC | AGCTCGACAATGTTCTCCAGC |
| *IRG1* | GCCAAGCTTCGACACGCTATA | AGTGGTTTCCTCCAGTGACCA |
| *HO1* | TATCGTGCTCGCAATGAACACTCTG | GTTGAGCAGGAAGGCGGTCTTAG |
| *NQO1* | AGAAGCGTCTGGAGACTGTCTGG | GATCTGGTTGTCGGCTGGAATGG |
| *GCLC* | GTCTCAAGAACATCGCCTCC | CTGCACATCTACCACGCAGT |
| *GCLM* | CATGTCCCATGCAGTGGAGAA | AGTCCAGCTGTGCAACTCCAA |
| *F4/80* | CGTGTTGTTGGTGGCACTGTGA | CCACATCAGTGTTCCAGGAGAC |
| *CD11c* | AAAATCTCCAACCCATGCTG | CACCACCAGGGTCTTCAAGT |

**Supplementary Table S3** The clinical description of the type 1 diabetes patients.

| Number | 1 | 2 | 3 | 4 | 5 | 6 | 7 | 8 | 9 | 10 |
| --- | --- | --- | --- | --- | --- | --- | --- | --- | --- | --- |
| Sex | M | M | W | M | M | M | M | M | W | M |
| Age (year) | 26 | 59 | 32 | 56 | 23 | 52 | 41 | 65 | 35 | 27 |
| BMI | 21.55 | 28.65 | 19.53 | 19.15 | 20.06 | 20.96 | 17.72 | 16.47 | 22.55 | 16.65 |
| Fasting blood glucose (mmol/L) | 19.84 | 15.22 | 17.57 | 13.39 | 12.9 | 17.12 | 11.06 | 8.98 | 7.63 | 7.05 |
| HbA1c (%) | 7.4 | 8.5 | 8.5 | 6.6 | 12.3 | 8 | 8.9 | 6.3 | 6.3 | 15.3 |
| Glycated Albumin (%) | 19.8 | 28.6 | 19.2 | 22.8 | 49.2 | 23.9 | 27.8 | 16 | 17.3 | 55.8 |
| Fasting C-peptide (nmol/L) | <3.33 | <3.33 | 6.7 | 15.7 | 108 | <3.33 | <3.33 | <3.33 | 89.4 | 43.4 |
| β-hydroxybutyric acid (mmol/L) | 0.3 | 0.54 | 1.06 | 0.14 | 2.07 | 0.06 | 0.34 | 0.18 | 0.02 | 2.79 |
| Autoimmunity antibodies | (-) | (-) | (-) | (+) | (-) | (-) | (+) | (-) | (-) | (+) |
| Systolic blood pressure (mmHg) | 140 | 120 | 92 | 131 | 133 | 109 | 94 | 99 | 114 | 123 |
| Diastolic blood pressure (mmHg) | 91 | 66 | 57 | 87 | 90 | 75 | 59 | 76 | 71 | 84 |
| Total cholesterol (mmol/L) | 6.18 | 4.6 | 3.54 | 4.23 | 4.53 | 5.48 | 4.77 | 3.13 | 5.24 | 2.84 |
| Triglycerides (mmol/L) | 2.87 | 1.1 | 0.58 | 0.84 | 1.04 | 0.99 | 6.99 | 0.55 | 0.87 | 0.68 |
| HDL cholesterol (mmol/L) | 1.18 | 1.54 | 1.51 | 2.16 | 0.81 | 1.3 | 0.86 | 1.14 | 1.8 | 0.9 |
| LDL cholesterol (mmol/L) | 5.02 | 2.63 | 1.8 | 1.94 | 2.95 | 3.68 | 2.7 | 1.66 | 2.83 | 1.64 |

**References**

1. Yi Z, Deng M, Scott MJ, Fu G, Loughran PA, Lei Z, Li S, Sun P, Yang C, Li W, et al. Immune-Responsive Gene 1/Itaconate Activates Nuclear Factor Erythroid 2-Related Factor 2 in Hepatocytes to Protect Against Liver Ischemia-Reperfusion Injury. Hepatology. 2020; 72(4): 1394-1411.
